# Cardiovascular Risk in Psoriatic Arthritis in the Treat-to-Target Era: A Focused Expert Narrative Review of Epidemiology, Risk Assessment, Vascular Imaging, and Prevention

**DOI:** 10.3390/jcm15187283

**Published:** 2026-09-19

**Authors:** Claudiu Avram, Cristiana Adina Avram, Maria-Laura Craciun, Ana-Maria Pah, Diana-Maria Mateescu, Daian Ionel Popa, Iasmina Isacov, Adina Duse

**Affiliations:** 1 Department XVI—Balneology, Medical Rehabilitation and Rheumatology, “Victor Babeș” University of Medicine and Pharmacy of Timișoara, Eftimie Murgu Square 2, 300041 Timișoara, Romania; avram.claudiu@umft.ro (C.A.); duse.adina@umft.ro (A.D.); 2 Transdisciplinary Research Center for Medical Rehabilitation, Balneology and Rheumatology (CTRMBR), “Victor Babeș” University of Medicine and Pharmacy of Timișoara, Eftimie Murgu Square 2, 300041 Timișoara, Romania; iasminaisacov@gmail.com; 3 Department of Internal Medicine I, “Victor Babeș” University of Medicine and Pharmacy of Timișoara, Eftimie Murgu Square 2, 300041 Timișoara, Romania; avram.adina@umft.ro; 4 Department of Cardiology, “Victor Babeș” University of Medicine and Pharmacy of Timișoara, Eftimie Murgu Square 2, 300041 Timișoara, Romania; 5 Department of Infectious Diseases, Municipal Emergency Clinical Hospital Timișoara, 2A Hector Street, 300041 Timișoara, Romania; diana.mateescu@umft.ro; 6 Research Center for Medical Communication, “Victor Babes” University of Medicine and Pharmacy, 300041 Timisoara, Romania; daian-ionel.popa@umft.ro; 7 Doctoral School, “Victor Babes” University of Medicine and Pharmacy of Timișoara, Eftimie Murgu Square 2, 300041 Timișoara, Romania

**Keywords:** psoriatic arthritis, cardiovascular disease, cardiovascular risk, atherosclerosis, metabolic syndrome, vascular imaging, risk prediction, treatment safety, prevention, narrative review

## Abstract

**Background/Objectives:** Psoriatic arthritis (PsA) is associated with excess cardiovascular (CV) morbidity, but the supporting literature spans heterogeneous observational, imaging, prediction, therapeutic, and guideline sources. This focused expert narrative review examines clinically relevant evidence for CV burden, risk markers, risk prediction, vascular imaging, treatment safety, and prevention in PsA. **Methods:** PubMed/MEDLINE was searched through 12 August 2026 using domain-based search blocks, supplemented by EULAR, GRAPPA, and European Society of Cardiology guidance and citation tracking. The final synthesis intentionally prioritized direct human PsA evidence; mixed psoriatic-disease or other immune-mediated evidence was used only when required for clinical context and was labeled as indirect. Embase, Scopus, and Web of Science were not searched; no formal risk-of-bias instrument or certainty-of-evidence grading framework was applied. **Results:** Conventional CV risk factors remain central. Direct PsA cohorts support associations between disease activity, metabolic abnormalities, and subsequent events, but do not establish incremental predictive value beyond validated risk models. Imaging studies demonstrate frequent subclinical atherosclerosis, yet plaque detection is distinct from event-prediction validation and does not establish benefit from universal screening. Therapeutic evidence is dominated by observational comparisons and low-event randomized-trial syntheses, precluding a causal cardioprotective ranking of drug classes. **Conclusions:** Current care should combine guideline-based CV prevention with treat-to-target PsA management for rheumatologic outcomes. PsA-specific risk modifiers, imaging-guided prevention, and treatment strategies intended to reduce hard CV outcomes require prospective validation. This article is a selective expert synthesis, not a systematic review, prediction model, or clinical pathway.

## 1. Introduction

Psoriatic arthritis (PsA) is a heterogeneous immune-mediated inflammatory disease encompassing peripheral arthritis, axial involvement, enthesitis, dactylitis, psoriasis, and nail disease, with a systemic burden extending beyond the musculoskeletal compartment [1]. Contemporary EULAR and GRAPPA recommendations require clinicians to consider extra-musculoskeletal manifestations and comorbidities when selecting therapy and defining long-term treatment goals [2,3]. Cardiometabolic disorders are among the most prevalent and clinically consequential of these comorbidities, and systematic evidence confirms that they occur more frequently in PsA than in healthy populations [4].

Cardiovascular disease (CVD) has therefore become an important component of comprehensive PsA care. Earlier systematic reviews documented increased cardiovascular morbidity [5], while more recent cohorts indicate that the magnitude of observed risk varies by calendar era, population characteristics, comorbidity burden, and treatment context. The clinically relevant questions are consequently narrower than whether PsA is simply “associated with CVD”: which risk signals are supported directly in PsA, whether they improve prediction beyond validated cardiovascular models, how vascular imaging should be interpreted, and what can—and cannot—be inferred from treatment-safety data.

Several recent reviews have already provided broad state-of-the-art, management-oriented, and mechanism-focused accounts of cardiovascular comorbidity in PsA [6,7,8]. The present review therefore restricts its scope to clinically relevant evidence on cardiovascular burden, conventional and PsA-associated risk markers, prediction, vascular imaging, treatment safety, and prevention. Myocardial fibrosis, cardiomyopathy, arrhythmias, venous thromboembolism, and experimental cytokine–cardiac pathways are not addressed because the available evidence is either indirect, mechanistic, or insufficiently PsA-specific for the clinical focus of this review.

The principal contribution is therefore a focused evidence map rather than a comprehensive appraisal of every cardiovascular phenotype. Direct PsA evidence is distinguished from mixed or indirect evidence, general cardiovascular evidence, formal guideline statements, and author interpretation. Throughout the review, association is separated from incremental prediction, detection of existing vascular disease from validation of future-event prediction, and treatment association from causal cardioprotection.

## 2. Methodological Approach, Focused Scope, and Evidence Provenance

This article was designed as a focused expert narrative review. Its purpose is selective clinical synthesis across a limited set of questions rather than exhaustive evidence retrieval, quantitative effect estimation, formal recommendation development, or certainty grading. The reporting approach was informed by the Scale for the Assessment of Narrative Review Articles (SANRA) [9]. The four review questions were: (1) what is the contemporary cardiovascular burden in PsA; (2) which conventional, inflammatory, and metabolic factors are clinically relevant risk markers and what predictive claims can they support; (3) how should validated risk models and vascular imaging be interpreted without conflating prediction with disease detection; and (4) what can reasonably be concluded about cardiovascular safety or benefit of PsA therapies?

PubMed/MEDLINE was searched through 12 August 2026 using domain-based search blocks for cardiovascular outcomes, inflammatory and cardiometabolic risk markers, vascular imaging, risk prediction, treatment safety, and review-level evidence. Exact executable search strings are provided in Supplementary Table S1. Searches were supplemented by official EULAR, GRAPPA, and European Society of Cardiology (ESC/ESC–EAS) guidance and backward citation tracking of pivotal PsA cohorts, meta-analyses, and contemporary reviews. Embase, Scopus, and Web of Science were not searched. Accordingly, the manuscript is explicitly positioned as a selective expert narrative synthesis rather than an exhaustive or systematic appraisal of all potentially eligible literature.

Search reporting and selection. The searches were conducted iteratively for a narrative review, and exact date-specific database yields were not prospectively archived when each search block was first run. To maximize reproducibility without presenting reconstructed historical totals as prospectively recorded data, Supplementary Table S1 reports the exact executable PubMed/MEDLINE strings with a fixed publication-date ceiling of 12 August 2026 and the number of cited sources retained for detailed synthesis within each domain. Retained-source counts are explicitly distinguished from database retrieval yields. Eligible evidence prioritized human PsA studies addressing clinical cardiovascular outcomes, conventional or disease-related risk markers, prediction, vascular imaging, or treatment safety. Mixed psoriasis/PsA evidence was retained only when directly relevant and when PsA-specific data were unavailable or not fully separable. Evidence from rheumatoid arthritis, psoriasis alone, other immune-mediated diseases, or experimental models was excluded from the core clinical synthesis except where needed to contextualize treatment safety or established general cardiovascular practice.

Structured interpretive appraisal. Study selection remained purposive, question-driven, and author-team based rather than a PRISMA-style dual-independent screening process, and no formal exclusion log was generated. For each major claim, the synthesis considered population directness, study design, sample size, outcome ascertainment, confounding, and the specific inference supported by the source. No formal risk-of-bias instrument or GRADE framework was applied. Evidence-provenance labels therefore describe where evidence comes from and how directly it applies to PsA; they are not grades of quality or certainty.

Claim-to-reference traceability. Major epidemiological, prognostic, imaging, therapeutic, and guideline-based statements were audited for source-to-claim fit. Supplementary Table S2 links each major claim to its evidence provenance, dominant study design, supporting references, and an explicit limit of inference. Independent adjusted association was not treated as evidence of incremental predictive utility unless discrimination, calibration, reclassification, or clinical utility was evaluated. Likewise, plaque detection was kept conceptually separate from event-prediction validation, and treatment associations were not translated into causal cardiovascular rankings without appropriate cardiovascular-endpoint evidence. The evidence-provenance categories used throughout the focused synthesis are summarized in Table 1.

## 3. Cardiovascular Burden in PsA

Historical observational evidence consistently identified increased cardiovascular morbidity in PsA. In the longitudinal Toronto cohort, Gladman and colleagues demonstrated excess cardiovascular morbidity compared with the general population [10]. A large UK population-based analysis subsequently reported increased major cardiovascular event rates in PsA even after adjustment for conventional risk factors [11]. A meta-analysis estimated approximately 43% higher overall cardiovascular morbidity and 22% higher cerebrovascular morbidity compared with the general population [12], while longitudinal follow-up from the Toronto cohort identified both traditional and disease-related factors as predictors of cardiovascular events [13].

The magnitude of excess risk is not static. A 2024 meta-analysis of longitudinal studies found that cardiovascular risk estimates in PsA declined across calendar time [14]. This secular trend is clinically relevant but does not identify which component of rheumatologic or cardiovascular care caused the decline. In a 2026 nationwide UK inception cohort of early inflammatory arthritis, cardiovascular hospitalization in PsA remained higher than in the general population (standardized incidence ratio [SIR] 1.26; 95% confidence interval [CI] 1.03–1.53), while hypertension, diabetes, and smoking were dominant associated factors [15].

Conversely, a large 2026 propensity-matched TriNetX analysis of 123,031 patients with PsA and an equal number of matched controls reported higher major adverse cardiovascular event (MACE) risk (hazard ratio [HR] 1.74), heart failure (HR 1.96), stroke (HR 1.49), and all-cause mortality (HR 1.95) [16]. These estimates should not be treated as contradictory to inception-cohort data; they illustrate the dependence of observed risk on age, comorbidity burden, disease duration, treatment exposure, healthcare setting, case definition, and outcome ascertainment. Residual confounding and code-based outcome misclassification remain important limitations of causal interpretation.

Prospective evidence also links inflammatory and metabolic phenotypes with subsequent cardiovascular events, but association should not be equated with incremental prediction. In the 10-year cardiovascular in rheumatology (CARMA) PsA cohort, the highest tertile of the Disease Activity Score in 28 joints using erythrocyte sedimentation rate (DAS28-ESR) remained independently associated with cardiovascular events after multivariable adjustment (HR 1.79; 95% CI 1.03–3.14), while marked hyperuricemia (urate ≥9.0 mg/dL) identified a higher-risk subgroup (HR 3.50; 95% CI 1.10–11.2) [17]. In a related analysis, a higher Metabolic Score for Insulin Resistance (METS-IR) was associated with later cardiovascular events [18]. Neither analysis demonstrated improved discrimination, calibration, reclassification, or clinical utility beyond a validated cardiovascular risk model. Representative studies defining cardiovascular burden and clinically relevant risk markers, together with their principal limits of inference, are summarized in Table 2.

## 4. Clinically Relevant Risk Architecture: Conventional, Metabolic, and Inflammatory Factors

**Figure 1:**
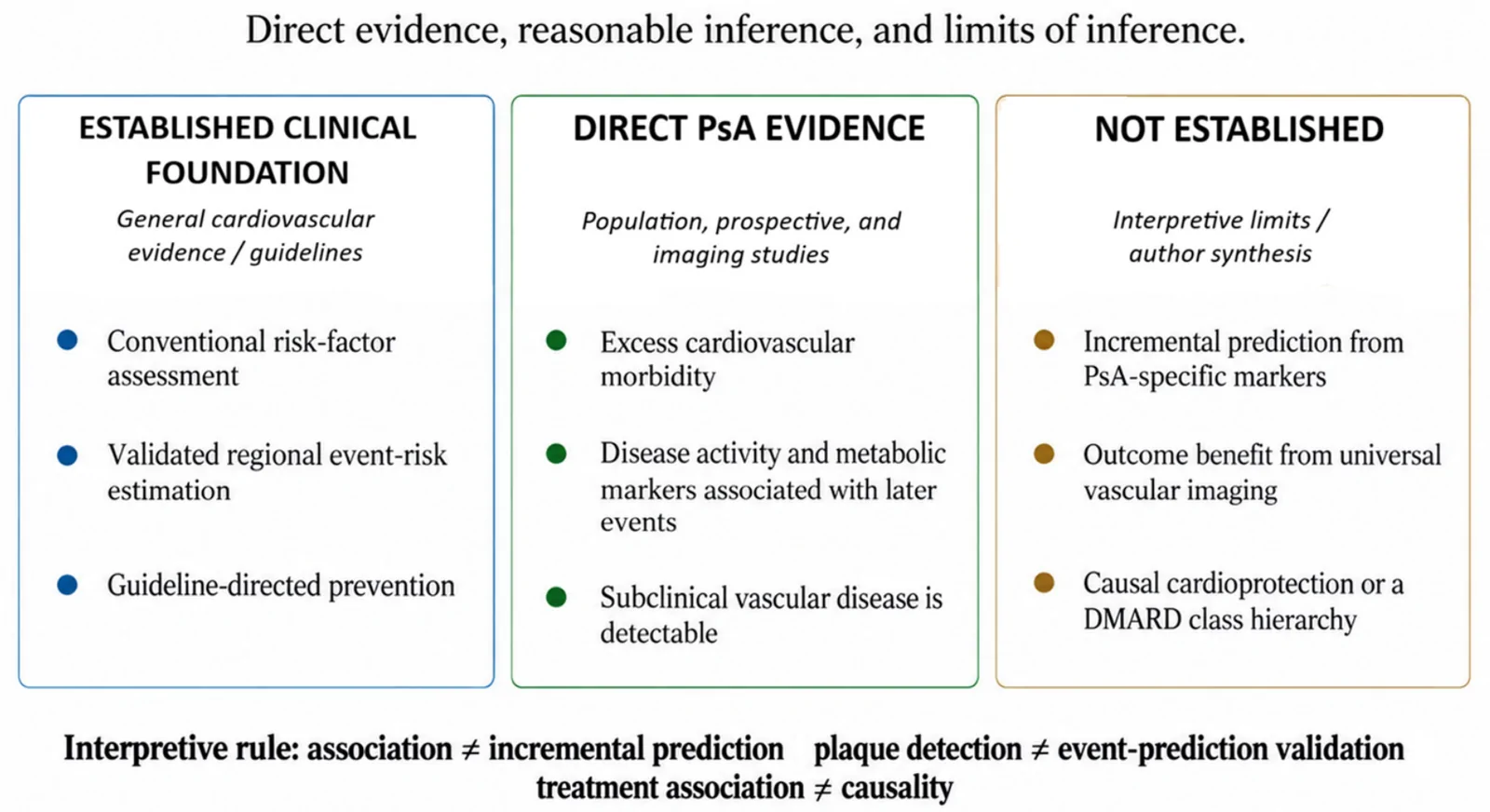
Focused evidence map for cardiovascular risk in psoriatic arthritis. The figure separates established general cardiovascular prevention, direct PsA associations or disease detection, and conclusions that are not established by the current evidence. It is an author-designed interpretive aid, not a causal model, prediction algorithm, or clinical pathway. Association does not imply incremental predictive utility; plaque detection does not validate future-event prediction; and treatment association does not establish causality. Figure 1 was created by the authors in Microsoft PowerPoint; no generative AI tools were used in its creation or modification.

Conventional cardiovascular risk factors remain the clinical foundation of risk assessment in PsA. Population-based PsA studies document a high burden and incomplete treatment of hypertension, dyslipidemia, diabetes, obesity, and smoking [19,20]. A meta-analysis estimated a pooled metabolic-syndrome prevalence of approximately 46% in PsA [21]. These findings support systematic cardiometabolic assessment, but they do not justify PsA-specific treatment thresholds that differ from established cardiovascular prevention guidance. These evidence domains and their corresponding limits of inference are summarized in Figure 1.

Inflammatory activity is clinically relevant, but the inferential boundary must remain explicit. Cumulative inflammatory burden has been associated with greater carotid plaque extent in PsA [22], and prospective data associate higher disease activity with later cardiovascular events [17]. Some vascular associations attenuate after adjustment for conventional risk factors [22], highlighting the potential roles of confounding and mediation. Current evidence therefore supports disease activity as a contextual risk marker, not as a validated quantitative modifier of cardiovascular risk.

Metabolic and inflammatory features frequently coexist. Obesity, insulin resistance, metabolic syndrome, and hyperuricemia may cluster with active disease and contribute to observed cardiovascular burden [18,21]. However, no PsA cardiovascular-endpoint intervention trial has established that targeting one of these markers specifically reduces MACE independently of standard cardiovascular risk-factor treatment and rheumatologic disease control.

Subclinical vascular abnormalities are discussed separately in Section 5 because they represent measured disease rather than a causal “hit” or a prediction model. Direct PsA imaging studies demonstrate that vascular abnormalities can be detected in patients without clinically evident CVD [23,24], but detection alone does not establish screening benefit or improve the predictive performance of a risk equation.

Figure 1 therefore organizes evidence according to what can be used clinically now, what is directly associated with cardiovascular burden in PsA, and what remains unproven. The simplified structure intentionally avoids causal arrows and mechanistic extrapolation.

## 5. Risk Prediction and Vascular Imaging: What the Evidence Supports

Population-derived cardiovascular risk equations estimate future events, whereas vascular imaging detects existing disease. These constructs should not be conflated. In psoriatic disease, the Framingham score has been shown to underestimate the extent of subclinical atherosclerosis in some imaging cohorts [25]. This demonstrates discordance between calculated risk and observed plaque burden; it does not by itself demonstrate poor discrimination or calibration for future cardiovascular events.

PsA-specific imaging studies have shown that carotid ultrasound can change risk categorization under imaging-informed clinical frameworks. Ultrasound-based reclassification has been associated with disease activity [26], and studies comparing multiple risk algorithms with carotid ultrasound have documented frequent score–plaque discordance [27,28]. These studies support the ability of imaging to reveal existing atherosclerosis, but they do not establish that adding imaging to a risk model improves future-event prediction or clinical outcomes.

Direct external validation is the appropriate test of prediction performance. In a 2024 validation study across inflammatory diseases, the QRISK3 cardiovascular risk algorithm, Framingham, and Reynolds models showed generally reasonable but lower discrimination in PsA than in their original general-population development settings [29]. This supports continued use of validated regional tools while recognizing imperfect transportability. It does not validate an untested PsA-specific correction factor.

The historical EULAR 1.5 multiplication factor was developed principally for rheumatoid arthritis under specified circumstances and should not be automatically transferred to PsA [30]. No universally accepted, externally validated PsA-specific multiplier or event-prediction equation currently exists.

Carotid ultrasound has the longest PsA-specific evidence base. Early studies documented a high prevalence of subclinical plaque even in patients without overt CVD or major conventional risk factors [23], and subsequent cohorts confirmed frequent carotid atherosclerosis [31]. More recent work suggests that carotid, femoral, and other vascular territories can provide non-identical information, including differences in diagnostic yield and cost-efficiency [32,33]. These studies establish feasibility and disease detection; they do not demonstrate incremental event prediction, net clinical benefit, or outcome improvement.

Coronary CT angiography and related coronary imaging can characterize coronary disease directly, and mixed psoriasis/PsA data suggest prognostic relevance of coronary atherosclerotic burden [34]. However, PsA-specific screening-outcome evidence remains insufficient to support routine coronary imaging solely because PsA is present.

Accordingly, vascular imaging is best framed as a selective decision modifier within established preventive cardiology practice, not as a test of whether a risk score has “failed”. Imaging should be considered only when it is consistent with general cardiovascular guidance and when the result is expected to change management [35,36]. Universal carotid, femoral, or coronary imaging of asymptomatic patients with PsA has not been shown to reduce cardiovascular events.

## 6. Antirheumatic Therapy and Cardiovascular Safety

Cardiovascular evidence for PsA therapies is heterogeneous and should be interpreted by study design. Most comparative signals come from observational cohorts, mixed psoriasis/PsA datasets, or meta-analyses of randomized trials in which cardiovascular events were uncommon and were not the primary endpoint. The appropriate objective is therefore cardiovascular safety interpretation, not construction of a cardioprotective hierarchy.

For NSAIDs and systemic glucocorticoids, caution is supported mainly by general cardiovascular and broader inflammatory-disease evidence rather than by PsA-specific cardiovascular-endpoint trials [37]. Potential blood-pressure, renal, metabolic, and cardiovascular effects justify minimization of unnecessary chronic exposure, but do not provide a PsA-specific comparative risk estimate. Observational data concerning methotrexate and TNF inhibitors are generally reassuring and in some analyses are associated with lower cardiovascular event rates or improved subclinical vascular indices [37,38,39]; these associations remain vulnerable to confounding by indication and disease control.

Randomized-trial syntheses provide a complementary but limited perspective. A meta-analysis of 77 randomized trials found no significant short-term increase in MACE or congestive heart failure among patients with psoriasis/PsA initiating biologic therapy [40]. A broader meta-analysis of targeted therapies likewise did not identify a simple class-wide cardiovascular toxicity signal [41]. Low event counts, short follow-up, and mixed populations mean that these analyses are more informative about absence of a large short-term safety signal than about long-term comparative cardioprotection.

Direct comparative observational evidence also requires caution. In a nationwide PsA cohort, weighted analyses reported differences in MACE hazards among some biologic classes and apremilast [42], while other large real-world analyses have reported modestly lower event rates among biologic DMARD users [16]. Channeling bias, residual confounding, treatment switching, and time-varying disease activity limit causal interpretation. These findings should not be converted into a therapeutic class ranking.

JAK inhibitors warrant explicit cardiovascular and thromboembolic risk assessment because the most influential randomized safety signal derives from ORAL Surveillance in rheumatoid arthritis, a cardiovascular-risk-enriched population [43]. This evidence is indirect for PsA. A PsA network meta-analysis has not demonstrated a definitive class-wide MACE excess, but event numbers remain low [44]. Current rheumatology guidance therefore supports individualized benefit–risk assessment rather than selection of a PsA therapy primarily for presumed cardioprotection [2,3,45].

Table 3 condenses the treatment evidence into three elements: the source of evidence, the inference that can reasonably be drawn, and the conclusion that the available evidence cannot support.

## 7. Clinical Interpretation Within Established Prevention

Current cardiovascular management in PsA should remain anchored to established prevention. Clinicians should assess smoking, blood pressure, adiposity, lipids, glycemia, kidney function, and personal or family cardiovascular history and use a validated regional risk equation when age-appropriate [35,36]. PsA diagnosis alone does not justify an automatic multiplication factor, and no PsA-specific equation has been externally validated for routine use [29,30].

Disease activity, repeated inflammatory-marker elevation, obesity, metabolic syndrome, hyperuricemia, and insulin resistance can provide clinically relevant context because direct PsA cohorts associate several of these features with later events [17,18,22]. However, they have not been shown to improve discrimination, calibration, reclassification, or clinical utility when added to a validated cardiovascular model. They should therefore prompt careful conventional risk assessment rather than unvalidated numerical adjustment.

Vascular imaging may be considered when a result is likely to resolve a genuine prevention decision and when its use is consistent with general cardiovascular guidance [35,36]. Evidence that plaque is frequent in PsA does not establish a universal screening program, nor does score–plaque discordance prove failure of an event-prediction model [23,26,27,28,29,30,31,32,33].

Intervention should prioritize evidence-based control of smoking, hypertension, dyslipidemia, diabetes, obesity, physical inactivity, and kidney disease according to established cardiovascular prevention guidance [35,36]. These actions are supported by general cardiovascular evidence and should not be presented as PsA-specific thresholds or targets.

Treat-to-target PsA management should be maintained because it is standard rheumatologic care [2,3]. Persistent disease activity is associated with adverse vascular phenotypes and later cardiovascular events in direct PsA data [17,22], but this association does not prove that treat-to-target therapy prevents MACE. Rheumatologic disease control and cardiovascular prevention should therefore be described as complementary rather than causally interchangeable strategies.

## 8. What Current Evidence Does Not Establish

First, current evidence does not prove that suppression of a specific PsA inflammatory pathway prevents myocardial infarction, stroke, or cardiovascular death. Observational associations between biologic therapy and cardiovascular outcomes remain vulnerable to confounding, while PsA randomized trials are not powered or long enough for definitive cardiovascular endpoint comparisons [16,37,38,39,40,41,42,43,44].

Second, the available evidence does not establish that one biologic or targeted synthetic class is universally superior for cardiovascular prevention. Comparative registry and administrative-database signals can inform safety surveillance and hypothesis generation but should not be converted into causal therapeutic rankings [40,41,42,43,44].

Third, PsA diagnosis, disease activity, inflammatory markers, metabolic markers, or imaging findings do not currently justify an unvalidated quantitative correction to a validated cardiovascular risk equation. Independent association and reclassification of observed plaque are not equivalent to demonstrated incremental prediction of future events [17,18,22,25,26,27,28,29,30].

Fourth, routine carotid, femoral, or coronary imaging of every asymptomatic patient with PsA has not been shown to reduce cardiovascular events. Plaque detection documents existing disease burden but does not by itself establish that population-wide imaging improves outcomes [23,26,27,28,29,30,31,32,33,34,35,36].

Finally, inflammatory control cannot replace conventional prevention. The association between lower disease activity and fewer cardiovascular events should not be interpreted as proof that treat-to-target therapy prevents MACE in PsA; contemporary data continue to show the central importance of hypertension, diabetes, smoking, adiposity, and dyslipidemia [15,17,18,19,20,21,22,35,36].

## 9. Research Priorities

Future research should prioritize prospective PsA cohorts with standardized cardiovascular outcome definitions, detailed conventional risk-factor measurement, repeated disease-activity assessment, and time-updated treatment exposure. Such cohorts are needed to quantify contemporary absolute risk and to distinguish markers of disease activity from independent predictors of cardiovascular events.

Any PsA-specific prediction model—or any proposal to add inflammatory, metabolic, or imaging variables to a general model—should be evaluated against contemporary validated tools using discrimination, calibration, reclassification, decision-curve analysis, external validation, and clinically meaningful risk thresholds. Association alone is insufficient for model adoption.

Vascular-imaging research should separate three questions: detection of existing plaque, incremental prediction of future events, and benefit from imaging-guided management. The most clinically important unanswered question is whether using imaging to change preventive treatment reduces hard cardiovascular outcomes, not simply whether plaque is more prevalent in PsA.

Comparative treatment research should use high-quality longitudinal registries with target-trial emulation and, where feasible, pragmatic randomized designs with prespecified cardiovascular endpoints. Analyses should account for confounding by indication, time-varying disease activity, prior therapy, baseline cardiovascular risk, smoking, weight change, and glucocorticoid or NSAID exposure.

Implementation research may offer the most immediate opportunity for benefit. Integrated rheumatology–primary-care or rheumatology–cardiology pathways should be evaluated for their ability to improve treatment of hypertension, LDL cholesterol, diabetes, obesity, smoking, and physical inactivity once these risks are identified [35,36].

## 10. Strengths and Limitations of This Review

This review has important limitations. It is a focused expert narrative synthesis rather than a systematic review. PubMed/MEDLINE, guideline repositories, and citation tracking were used, but Embase, Scopus, Web of Science, grey literature, and formal duplicate screening were not included. Exact date-specific search yields were not prospectively archived during the original iterative search process; the supplement therefore provides exact executable queries with a fixed publication-date ceiling and clearly distinguishes retained-source counts from retrieval yields. No formal risk-of-bias instrument or certainty-of-evidence framework was applied. These choices limit comprehensiveness, reproducibility, and the strength of any framework-like inference. Accordingly, the review does not claim exhaustive coverage, formal evidence grading, or guideline authority.

Its main strength is a tighter alignment between methodology and claims. The scope prioritizes clinically relevant direct PsA evidence and excludes extended myocardial, arrhythmic, and mechanistic extrapolation. Evidence provenance is separated from study design; major claims are linked to proximate citations and explicit limits of inference in Supplementary Table S2; association is separated from incremental prediction; plaque detection is separated from event-prediction validation; and treatment associations are not presented as causal cardioprotection. These features improve transparency without converting a narrative review into a systematic evidence synthesis.

## 11. Conclusions

Psoriatic arthritis is associated with clinically important cardiovascular morbidity and a high burden of conventional cardiometabolic risk factors. Direct PsA studies also support associations between disease activity, metabolic abnormalities, and later cardiovascular events, as well as frequent subclinical atherosclerosis. However, these observations do not by themselves establish incremental prediction, justify a PsA-specific risk multiplier, support universal vascular imaging, or prove that one antirheumatic drug class prevents cardiovascular events better than another.

Current management should therefore remain anchored to established evidence: apply validated regional cardiovascular risk tools; treat smoking, hypertension, dyslipidemia, diabetes, obesity, physical inactivity, and kidney disease according to general cardiovascular guidance; and maintain treat-to-target PsA therapy for rheumatologic outcomes. Selective vascular imaging may be appropriate when consistent with general prevention guidance and when the result is expected to change management.

The principal unresolved questions are prospective: whether PsA-specific variables improve event prediction, whether imaging-guided prevention improves outcomes, and whether any treatment strategy reduces hard cardiovascular events independently of conventional prevention and disease-control effects. Until those questions are answered, the most defensible approach is rigorous application of established cardiovascular prevention combined with careful, evidence-bounded interpretation of PsA-specific data.

## Figures and Tables

**Table 1:** Evidence-provenance framework used in the focused synthesis. Categories indicate source applicability and intended interpretive use, not methodological quality or certainty.

Evidence Source Category	Definition/Applicability	Appropriate Use in This Review	Interpretive Limitation
Direct PsA evidence	Population and cardiovascular outcome or marker are explicitly reported for PsA.	Primary basis for PsA-specific epidemiology, association, prediction, imaging, and treatment-safety statements.	Directness does not remove confounding, measurement error, limited sample size, or other design-specific bias.
Mixed/indirect inflammatory-disease evidence	Psoriasis and PsA are combined, or evidence derives from another immune-mediated inflammatory disease.	Used selectively when clinically relevant PsA-specific evidence is unavailable, especially for safety context.	Cannot establish a PsA-specific effect size, mechanism, or therapeutic ranking.
General cardiovascular evidence	Evidence derives from general-population prevention, prediction, or cardiovascular treatment studies.	Supports established management of blood pressure, lipids, diabetes, smoking, obesity, and use of validated risk tools.	Methodologically strong evidence may still be non-PsA-specific.
Guideline-derived evidence	Formal recommendations from professional societies.	Defines established rheumatology or cardiovascular practice within the scope of the guideline.	Recommendation strength is inherited from the source guideline and is not regraded here.
Author interpretation	Integration of the preceding evidence categories by the authors.	Used to clarify boundaries, unresolved questions, and clinically cautious interpretation.	Conceptual only; not a guideline, prediction model, or validated clinical pathway.

**Table 2:** Representative PsA studies defining cardiovascular burden and clinically relevant risk markers, with explicit limits of inference.

Study	Evidence Provenance + Design	Key Result	Interpretive Boundary
Polachek et al., 2017 [12]	Direct PsA; meta-analysis of observational studies	~43% higher cardiovascular morbidity and ~22% higher cerebrovascular morbidity.	Historical pooled association; heterogeneity and calendar era limit use as a contemporary individual-risk estimate.
Gouze et al., 2024 [14]	Direct PsA; meta-analysis of longitudinal studies	Cardiovascular risk estimates declined across calendar time.	Supports a secular trend; does not identify which treatment or prevention component caused the change.
Yang et al., 2026 [15]	Direct PsA within an early inflammatory-arthritis inception cohort	PsA CVD hospitalization SIR 1.26 (95% CI 1.03–1.53); conventional factors were dominant.	Contemporary association; not a PsA-specific prediction model or proof that treat-to-target prevents CVD.
Tyczyńska et al., 2026 [16]	Direct PsA; retrospective federated propensity-matched cohort	MACE HR 1.74; HF HR 1.96; stroke HR 1.49 versus matched controls.	Large real-world signal; code-based outcomes and residual confounding limit causal inference, especially for treatment comparisons.
Llorca et al., 2026 [17]	Direct PsA; 10-year prospective cohort	Higher disease activity and marked hyperuricemia were independently associated with later CV events.	Independent association does not establish incremental discrimination, calibration, reclassification, or clinical utility.
González-Gay et al., 2025 [18]	Direct PsA; 10-year prospective metabolic analysis	Higher METS-IR was associated with later cardiovascular events.	Supports a metabolic risk-marker phenotype; incremental predictive value beyond validated scores remains unproven.

**Table 3:** Cardiovascular interpretation of major PsA treatment categories: evidence source, reasonable inference, and unsupported conclusion.

Evidence Source	Reasonable Inference	Conclusion not Supported
NSAIDs/systemic glucocorticoids—general cardiovascular and broader inflammatory-disease evidence [35,37]	Potential blood-pressure, renal, metabolic, and cardiovascular effects justify minimizing unnecessary chronic exposure.	A PsA-specific MACE estimate or comparative cardiovascular ranking.
Methotrexate/TNF inhibitors—predominantly observational mixed or direct PsA evidence [16,37,38,39,42]	Overall signals are reassuring; some cohorts and imaging studies report lower event rates or more favorable vascular indices.	Causal cardioprotection, a cardiovascular indication, or superiority over other effective PsA therapies.
IL-17/IL-12/23/IL-23 inhibitors—mixed psoriasis/PsA randomized-trial syntheses plus comparative observational PsA data [40,41,42]	No large short-term MACE safety signal has been established; comparative observational findings are heterogeneous.	Long-term cardiovascular equivalence, harm, or a causal class hierarchy.
Apremilast—comparative observational PsA evidence [42]	No clear excess MACE signal has been established in available comparative data.	Cardiovascular protection or superiority to biologic classes.
JAK inhibitors—direct low-event PsA trials/meta-analysis plus extrapolated RA safety RCT [43,44]	Individualized cardiovascular and thromboembolic risk assessment is appropriate, particularly in higher-risk patients.	Direct transfer of RA effect sizes to PsA or a universal PsA class ranking.

## Data Availability

No new data were generated or analyzed in this study. All information is derived from previously published studies, which are cited within the manuscript.

## Supplementary Materials

The following supporting information can be downloaded at https://www.mdpi.com/article/10.3390/jcm15187283/s1, Supplementary Table S1: Reproducible PubMed/MEDLINE search blocks for the focused review, with a fixed publication-date ceiling of 12 August 2026, domain use, and retained-source counts; Supplementary Table S2: Claim-to-evidence audit for the major clinically relevant statements retained in the review. For each claim, the table records the evidence source/provenance and dominant study design, the corresponding reference numbers in the manuscript, and the limit of inference. Provenance indicates applicability and source type, not methodological quality or certainty; no formal risk-of-bias or certainty-grading framework was applied.

## Abbreviations

The following abbreviations are used in this manuscript:

CARMA	CARdiovascular in RheuMAtology
CI	Confidence interval
CT	Computed tomography
CV	Cardiovascular
CVD	Cardiovascular disease
DAS28-ESR	Disease Activity Score in 28 joints using erythrocyte sedimentation rate
DMARD	Disease-modifying antirheumatic drug
EAS	European Atherosclerosis Society
ESC	European Society of Cardiology
ESR	Erythrocyte sedimentation rate
EULAR	European Alliance of Associations for Rheumatology
GRAPPA	Group for Research and Assessment of Psoriasis and Psoriatic Arthritis
HF	Heart failure
HR	Hazard ratio
IL	Interleukin
JAK	Janus kinase
LDL	Low-density lipoprotein
MACE	Major adverse cardiovascular events
METS-IR	Metabolic Score for Insulin Resistance
NSAID	Non-steroidal anti-inflammatory drug
PsA	Psoriatic arthritis
QRISK3	QRISK3 cardiovascular risk prediction algorithm
RA	Rheumatoid arthritis
RCT	Randomized controlled trial
SANRA	Scale for the Assessment of Narrative Review Articles
SIR	Standardized incidence ratio
TNF	Tumor necrosis factor

## References

[1] FitzGerald O., Ogdie A., Chandran V., Coates L.C., Kavanaugh A., Tillett W., Leung Y.Y., de Wit M., Scher J.U., Mease P.J. (2021). Psoriatic arthritis. Nat. Rev. Dis. Primers.

[2] Gossec L., Kerschbaumer A., Ferreira R.J.O., Aletaha D., Baraliakos X., Bertheussen H., Boehncke W.-H., Esbensen B.A., McInnes I.B., McGonagle D. (2024). EULAR recommendations for the management of psoriatic arthritis with pharmacological therapies: 2023 update. Ann. Rheum. Dis..

[3] Coates L.C., Soriano E.R., Corp N., Bertheussen H., Callis Duffin K., Campanholo C.B., Chau J., Eder L., Fernández-Ávila D.G., FitzGerald O. (2022). Group for Research and Assessment of Psoriasis and Psoriatic Arthritis (GRAPPA): Updated treatment recommendations for psoriatic arthritis 2021. Nat. Rev. Rheumatol..

[4] Gupta S., Syrimi Z., Hughes D.M., Zhao S.S. (2021). Comorbidities in psoriatic arthritis: A systematic review and meta-analysis. Rheumatol. Int..

[5] Jamnitski A., Symmons D., Peters M.J.L., Sattar N., McInnes I., Nurmohamed M.T. (2013). Cardiovascular comorbidities in patients with psoriatic arthritis: A systematic review. Ann. Rheum. Dis..

[6] Dey M., Nikiphorou E. (2024). Cardiovascular comorbidities in psoriatic arthritis: State of the art. Ther. Adv. Musculoskelet. Dis..

[7] Atzeni F., Rodríguez-Carrio J., Alciati A., Tropea A., Marchesoni A. (2025). Cardiovascular risk in psoriatic arthritis: How can we manage it?. Autoimmun. Rev..

[8] Totolici S., Vrabie A.M., Buzea C.A., Badila E. (2026). Cardiovascular risk in psoriatic arthritis: Mechanisms, risk assessment, and long-term management implications. Int. J. Mol. Sci..

[9] Baethge C., Goldbeck-Wood S., Mertens S. (2019). SANRA—A scale for the quality assessment of narrative review articles. Res. Integr. Peer Rev..

[10] Gladman D.D., Ang M., Su L., Tom B.D.M., Schentag C.T., Farewell V.T. (2009). Cardiovascular morbidity in psoriatic arthritis. Ann. Rheum. Dis..

[11] Ogdie A., Yu Y., Haynes K., Love T.J., Maliha S., Jiang Y., Troxel A.B., Hennessy S., Kimmel S.E., Margolis D.J. (2015). Risk of major cardiovascular events in patients with psoriatic arthritis, psoriasis and rheumatoid arthritis: A population-based cohort study. Ann. Rheum. Dis..

[12] Polachek A., Touma Z., Anderson M., Eder L. (2017). Risk of cardiovascular morbidity in patients with psoriatic arthritis: A meta-analysis of observational studies. Arthritis Care Res..

[13] Eder L., Wu Y., Chandran V., Cook R.J., Gladman D.D. (2016). Incidence and predictors for cardiovascular events in patients with psoriatic arthritis. Ann. Rheum. Dis..

[14] Gouze H., Aegerter P., Gouyette Y., Breban M., D’Agostino M.A. (2024). Risk of cardiovascular disease decreases over time in psoriatic arthritis but not in spondylarthritis: Meta-analysis of longitudinal studies. Rheumatology.

[15] Yang Z., Atzeni F., Russell M., Song K., Coalwood C., Price E., Buch M., Norton S., Galloway J. (2026). Patterns and risk of cardiovascular disease in rheumatoid arthritis and psoriatic arthritis: A nationwide cohort study in the UK. Rheumatol. Adv. Pract..

[16] Tyczyńska K., Krajewski P.K., Złotowska A., Szepietowski J.C., Świerkot J. (2026). Excess cardiovascular morbidity in psoriatic arthritis and cardioprotective effects of biologic DMARDs: A propensity-matched analysis. Sci. Rep..

[17] Llorca J., Ferraz-Amaro I., Moreno-Martínez M.J., Plaza Z., Sánchez-Alonso F., Moreno-Ramos M.J., García-Gómez C., Castañeda S., González-Juanatey C., González-Gay M.Á. (2026). Disease activity and hyperuricemia predict the development of cardiovascular events in patients with psoriatic arthritis: Results of the 10-year prospective evaluation in the CARMA cohort. Semin. Arthritis Rheum..

[18] González-Gay M.A., Ferraz-Amaro I., Castañeda S., Pinto-Tasende J.A., Uriarte-Ecenarro M., Plaza Z., Sánchez-Alonso F., García-Gómez C., González-Juanatey C., Llorca J. (2025). The metabolic score for insulin resistance predicts the risk of cardiovascular disease in patients with psoriatic arthritis: Results from the 10-year prospective CARMA cohort. RMD Open.

[19] Jafri K., Bartels C.M., Shin D., Gelfand J.M., Ogdie A. (2017). Incidence and management of cardiovascular risk factors in psoriatic arthritis and rheumatoid arthritis: A population-based study. Arthritis Care Res..

[20] Gulati A.M., Semb A.G., Rollefstad S., Romundstad P.R., Kavanaugh A., Gulati S., Haugeberg G., Hoff M. (2016). On the HUNT for cardiovascular risk factors and disease in patients with psoriatic arthritis: Population-based data from the Nord-Trøndelag Health Study. Ann. Rheum. Dis..

[21] Loganathan A., Kamalaraj N., El-Haddad C., Pile K. (2021). Systematic review and meta-analysis on prevalence of metabolic syndrome in psoriatic arthritis, rheumatoid arthritis and psoriasis. Int. J. Rheum. Dis..

[22] Eder L., Thavaneswaran A., Chandran V., Cook R., Gladman D.D. (2015). Increased burden of inflammation over time is associated with the extent of atherosclerotic plaques in patients with psoriatic arthritis. Ann. Rheum. Dis..

[23] Gonzalez-Juanatey C., Llorca J., Amigo-Diaz E., Dierssen T., Martin J., Gonzalez-Gay M.A. (2007). High prevalence of subclinical atherosclerosis in psoriatic arthritis patients without clinically evident cardiovascular disease or classic atherosclerosis risk factors. Arthritis Rheum..

[24] Di Minno M.N.D., Ambrosino P., Lupoli R., Di Minno A., Tasso M., Peluso R., Tremoli E. (2015). Cardiovascular risk markers in patients with psoriatic arthritis: A meta-analysis of literature studies. Ann. Med..

[25] Eder L., Chandran V., Gladman D.D. (2014). The Framingham Risk Score underestimates the extent of subclinical atherosclerosis in patients with psoriatic disease. Ann. Rheum. Dis..

[26] Palmou-Fontana N., Martínez-López D., Corrales A., Rueda-Gotor J., Genre F., Armesto S., González-López M.A., Quevedo-Abeledo J.C., Portilla-González V., Blanco R. (2020). Disease activity influences cardiovascular risk reclassification based on carotid ultrasound in patients with psoriatic arthritis. J. Rheumatol..

[27] Galarza-Delgado D.A., Azpiri-López J.R., Colunga-Pedraza I.J., Guajardo-Jauregui N., Rodriguez-Romero A.B., Lugo-Perez S., Cardenas-de la Garza J.A., Arvizu-Rivera R.I., Flores-Alvarado D.E., Ilizaliturri-Guerra O. (2022). Cardiovascular risk reclassification according to six cardiovascular risk algorithms and carotid ultrasound in psoriatic arthritis patients. Clin. Rheumatol..

[28] Martínez-Vidal M.P., Andrés M., Jovani V., Santos-Ramírez C., Romera C., Fernández-Carballido C. (2020). Role of carotid ultrasound and Systematic Coronary Risk Evaluation charts for the cardiovascular risk stratification of patients with psoriatic arthritis. J. Rheumatol..

[29] Hughes D.M., Cuitun Coronado J.I., Schofield P., Yiu Z.Z.N., Zhao S.S. (2024). The predictive accuracy of cardiovascular disease risk prediction tools in inflammatory arthritis and psoriasis: An observational validation study using the Clinical Practice Research Datalink. Rheumatology.

[30] Agca R., Heslinga S.C., Rollefstad S., Heslinga M., McInnes I.B., Peters M.J.L., Kvien T.K., Dougados M., Radner H., Atzeni F. (2017). EULAR recommendations for cardiovascular disease risk management in patients with rheumatoid arthritis and other forms of inflammatory joint disorders: 2015/2016 update. Ann. Rheum. Dis..

[31] Ibáñez-Bosch R., Restrepo-Velez J., Medina-Malone M., Garrido-Courel L., Paniagua-Zudaire I., Loza-Cortina E. (2017). High prevalence of subclinical atherosclerosis in psoriatic arthritis patients: A study based on carotid ultrasound. Rheumatol. Int..

[32] Charca L.C., Queiro R. (2026). Cost-efficiency of imaging strategies for detecting subclinical atherosclerosis in psoriatic arthritis. Clin. Rheumatol..

[33] Charca L.C., Braña I., Loredo M., Alvarez P., Pardo E., Burger S., Queiro R. (2026). Multisite atherosclerosis and SCORE2-based risk stratification in psoriatic arthritis: A phenotype-dependent role of vascular territories. Biomedicines.

[34] Tinggaard A.B., Hjuler K.F., Andersen I.T., Winther S., Iversen L., Bøttcher M. (2021). Prevalence and severity of coronary artery disease linked to prognosis in psoriasis and psoriatic arthritis patients: A multi-centre cohort study. J. Intern. Med..

[35] Visseren F.L.J., Mach F., Smulders Y.M., Carballo D., Koskinas K.C., Bäck M., Benetos A., Biffi A., Boavida J.M., Capodanno D. (2021). 2021 ESC Guidelines on cardiovascular disease prevention in clinical practice. Eur. Heart J..

[36] Mach F., Koskinas K.C., Roeters van Lennep J.E., Tokgözoğlu L., Badimon L., Baigent C., Benn M., Binder C.J., Catapano A.L., De Backer G.G. (2025). 2025 Focused Update of the 2019 ESC/EAS Guidelines for the management of dyslipidaemias. Eur. Heart J..

[37] Roubille C., Richer V., Starnino T., McCourt C., McFarlane A., Fleming P., Siu S., Kraft J., Lynde C., Pope J. (2015). The effects of tumour necrosis factor inhibitors, methotrexate, non-steroidal anti-inflammatory drugs and corticosteroids on cardiovascular events in rheumatoid arthritis, psoriasis and psoriatic arthritis: A systematic review and meta-analysis. Ann. Rheum. Dis..

[38] Yang Z.S., Lin N.N., Li L., Li Y. (2016). The effect of TNF inhibitors on cardiovascular events in psoriasis and psoriatic arthritis: An updated meta-analysis. Clin. Rev. Allergy Immunol..

[39] Eder L., Joshi A.A., Dey A.K., Cook R., Siegel E.L., Gladman D.D., Mehta N.N. (2018). Association of tumor necrosis factor inhibitor treatment with reduced indices of subclinical atherosclerosis in patients with psoriatic disease. Arthritis Rheumatol..

[40] Champs B., Degboé Y., Barnetche T., Cantagrel A., Ruyssen-Witrand A., Constantin A. (2019). Short-term risk of major adverse cardiovascular events or congestive heart failure in patients with psoriatic arthritis or psoriasis initiating a biological therapy: A meta-analysis of randomised controlled trials. RMD Open.

[41] Cai R., Jin Y., Chen B., Zhao J., Zhai J., Zeng L., Mu R. (2023). Impact of targeted therapies on the risk of cardiovascular events in patients with psoriasis and psoriatic arthritis: A systematic review and aggregate data meta-analysis of randomized controlled trials. Int. J. Rheum. Dis..

[42] Pina Vegas L., Le Corvoisier P., Penso L., Paul M., Sbidian E., Claudepierre P. (2022). Risk of major adverse cardiovascular events in patients initiating biologics/apremilast for psoriatic arthritis: A nationwide cohort study. Rheumatology.

[43] Ytterberg S.R., Bhatt D.L., Mikuls T.R., Koch G.G., Fleischmann R., Rivas J.L., Germino R., Menon S., Sun Y., Wang C. (2022). Cardiovascular and cancer risk with tofacitinib in rheumatoid arthritis. N. Engl. J. Med..

[44] Shi L.H., Wei J.C.C., Tung W.T.H. (2025). Risk of major adverse cardiovascular events and thromboembolism events in patients with psoriatic arthritis on JAK inhibitors: A network meta-analysis. Rheumatol. Ther..

[45] Campanholo C.B., Maharaj A.B., Corp N., Bell S., Costa L., de Vlam K., Gullick N.J., Khraishi M., Kishimoto M., Palmou-Fontana N. (2023). Management of psoriatic arthritis in patients with comorbidities: An updated literature review informing the 2021 GRAPPA treatment recommendations. J. Rheumatol..

