# Supplementary material for: Cardiovascular Risk in Psoriatic Arthritis in the Treat-to-Target Era: A Focused Expert Narrative Review of Epidemiology, Risk Assessment, Vascular Imaging, and Prevention
Source: J Clin Med. 2026 Sep 19;15(18):7283. doi: 10.3390/jcm15187283 (PMC13608499; doi:10.3390/jcm15187283)
Supplement: Supplementary file 1 [file jcm-15-07283-s001.zip › jcm-4542872-supplementary.pdf]

Supplementary Methods S1 and Tables S1–S2

This supplement makes the PubMed/MEDLINE component of the focused expert narrative review auditable. The search strings below correspond to the operational domains retained in the manuscript and are reported for reproducibility. The review was designed for selective clinical synthesis rather than exhaustive systematic retrieval, quantitative pooling, or guideline development. Searches were current through 12 August 2026; official EULAR, GRAPPA, and ESC/EAS repositories and backward citation tracking were used in addition to PubMed/MEDLINE.

Database limitation. Embase, Scopus, and Web of Science were not searched. The manuscript therefore does not claim exhaustive coverage of all potentially eligible literature and is explicitly positioned as a focused expert narrative synthesis.

Search-yield reporting. The searches were conducted iteratively for a narrative review, and exact date-specific PubMed retrieval counts were not prospectively archived when each historical search block was first run. To maximize reproducibility without presenting reconstructed historical totals as prospectively recorded data, every executable query in Table S1 includes the fixed publication-date restriction ("1900/01/01"[dp] : "2026/08/12"[dp]). Table S1 separately reports the number of cited sources retained for detailed synthesis in each domain. Retained-source counts are not database retrieval yields, may overlap across domains, and should not be interpreted as a PRISMA-style study flow.

Table S1. Reproducible PubMed/MEDLINE search blocks for the focused review, with a fixed publication-date ceiling of 12 August 2026, domain use, and retained-source counts.

Domain	Reproducible PubMed/MEDLINE search block (publication dates through 12 August 2026)	Use in synthesis	Cited sources retained*
Core cardiovascular outcomes	("Arthritis, Psoriatic"[Mesh] OR "psoriatic arthritis"[tiab]) AND ("Cardiovascular Diseases"[Mesh] OR cardiovascular[tiab] OR "major adverse cardiovascular"[tiab] OR MACE[tiab] OR "myocardial infarction"[tiab] OR stroke[tiab] OR "heart failure"[tiab]) AND ("1900/01/01"[dp] : "2026/08/12"[dp])	Contemporary/historical cardiovascular burden and event outcomes.	7 (refs. 10–16)
Inflammatory and cardiometabolic phenotype	("Arthritis, Psoriatic"[Mesh] OR "psoriatic arthritis"[tiab]) AND (inflamm*[tiab] OR CRP[tiab] OR ESR[tiab] OR "disease activity"[tiab] OR obesity[tiab] OR "metabolic syndrome"[tiab] OR "insulin resistance"[tiab] OR METS-IR[tiab] OR "uric acid"[tiab] OR hyperuricemia[tiab]) AND (cardiovascular[tiab] OR atherosclero*[tiab] OR MACE[tiab]) AND ("1900/01/01"[dp] : "2026/08/12"[dp])	Disease-activity and metabolic risk-marker associations.	6 (refs. 17–22)
Vascular imaging and subclinical disease	("Arthritis, Psoriatic"[Mesh] OR "psoriatic arthritis"[tiab]) AND (atherosclero*[tiab] OR carotid[tiab] OR femoral[tiab] OR "intima-media thickness"[tiab] OR plaque[tiab] OR "coronary artery calcium"[tiab] OR "coronary computed tomography angiography"[tiab]) AND ("1900/01/01"[dp] : "2026/08/12"[dp])	Carotid/femoral imaging, cIMT, plaque, CAC/CTA, and multisite disease.	10 (refs. 23–28, 31–34)
Risk prediction and reclassification	("Arthritis, Psoriatic"[Mesh] OR "psoriatic arthritis"[tiab]) AND ("risk prediction"[tiab] OR "risk score"[tiab] OR SCORE2[tiab] OR QRISK[tiab] OR Framingham[tiab] OR Reynolds[tiab] OR reclassification[tiab] OR calibration[tiab] OR discrimination[tiab]) AND ("1900/01/01"[dp] : "2026/08/12"[dp])	Prediction-model validation and imaging-related reclassification.	6 (refs. 25–30)

Domain	Reproducible PubMed/MEDLINE search block (publication dates through 12 August 2026)	Use in synthesis	Cited sources retained*
Treatment and cardiovascular safety	("Arthritis, Psoriatic"[Mesh] OR "psoriatic arthritis"[tiab]) AND (DMARD[tiab] OR biologic*[tiab] OR "TNF inhibitor"[tiab] OR IL-17[tiab] OR IL-23[tiab] OR JAK[tiab] OR methotrexate[tiab] OR apremilast[tiab] OR NSAID[tiab] OR glucocorticoid*[tiab]) AND (cardiovascular[tiab] OR MACE[tiab] OR "heart failure"[tiab] OR "myocardial infarction"[tiab] OR stroke[tiab] OR thromboembol*[tiab]) AND ("1900/01/01"[dp] : "2026/08/12"[dp])	Treatment-associated cardiovascular safety and comparative observational signals.	9 (refs. 37–45)
Reviews and evidence syntheses	("Arthritis, Psoriatic"[Mesh] OR "psoriatic arthritis"[tiab]) AND (cardiovascular[tiab] OR atheroscler0*[tiab]) AND (systematic review[pt] OR meta-analysis[pt] OR review[pt]) AND ("1900/01/01"[dp] : "2026/08/12"[dp])	Contextualization, prior-review comparison, and backward citation tracking.	5 core contextual reviews (refs. 4–8)

*Retained-source counts describe cited sources used in the final synthesis, not database retrieval yields; sources may contribute to more than one domain. Professional guidance was additionally identified in official EULAR, GRAPPA, and ESC/EAS repositories. Some contextual sources that cannot be retrieved by a PsA-specific search block—for example ORAL Surveillance in rheumatoid arthritis [43]—were identified through backward citation tracking and/or guideline-linked citation tracking and were used only as explicitly labeled indirect safety context.

Eligibility and prioritization rules. Human PsA evidence was prioritized for cardiovascular epidemiology, clinically relevant risk markers, prediction, vascular imaging, and treatment safety. Mixed psoriasis/PsA evidence was retained only when directly relevant and PsA-specific results were not fully separable. Evidence from rheumatoid arthritis or other immune-mediated inflammatory diseases was used sparingly for safety context and was explicitly labeled as indirect. Extended myocardial-fibrosis, cardiomyopathy, arrhythmia, venous-thromboembolism, and experimental cytokine-cardiac pathways are outside the scope of this focused review because their evidentiary base is too indirect for the clinical purpose. Current EULAR, GRAPPA, and ESC/EAS guidance was searched separately in official repositories. Backward and guideline-linked citation tracking was used for contextual sources not captured by PsA-specific PubMed blocks, including the RA-only ORAL Surveillance safety trial [43]. Selection was author-team based, purposive, question-driven, and iterative; no PRISMA-style dual-independent screening or formal exclusion log was generated. No formal risk-of-bias instrument or GRADE certainty assessment was applied.

Table S2. Claim-to-evidence audit for the major clinically relevant statements retained in the review. For each claim, the table records the evidence source/provenance and dominant study design, the corresponding reference numbers in the manuscript, and the limit of inference. Provenance indicates applicability and source type, not methodological quality or certainty; no formal risk-of-bias or certainty-grading framework was applied.

Section	**Major claim retained in the review**	Evidence source/provenance and dominant design [ref.]	Limit of inference
3	PsA is associated with excess cardiovascular and cerebrovascular morbidity.	Direct PsA; population cohorts and meta-analyses of observational studies [10–13,16].	Association and historical pooled burden; not proof of a PsA-specific causal pathway or an individual prediction estimate.
3	Excess cardiovascular risk estimates have declined across calendar time.	Direct PsA; meta-analysis of longitudinal studies [14].	Secular trend only; the responsible rheumatologic or cardiovascular-care component is not identified.

Section	**Major claim retained in the review**	Evidence source/provenance and dominant design [ref.]	Limit of inference
3	Cardiovascular hospitalization remains higher than in the general population in a contemporary inception cohort.	Direct PsA within an early inflammatory-arthritis inception cohort [15].	Association in a defined health system; not a PsA-specific prediction model and not evidence that treat-to-target prevents events.
3	Disease activity, marked hyperuricemia, and METS-IR are independently associated with later cardiovascular events.	Direct PsA; prospective CARMA cohort [17,18].	Independent association does not establish incremental discrimination, calibration, reclassification, or clinical utility.
4	Conventional risk factors are common and incompletely treated in PsA.	Direct PsA; population-based observational studies [19,20].	Supports systematic assessment; does not establish a PsA-specific treatment threshold.
4	Metabolic syndrome and metabolic dysfunction are highly prevalent in PsA.	Direct PsA; systematic review/meta-analysis and prospective association data [18,21].	Prevalence/association only; no PsA cardiovascular-endpoint intervention effect is established.
4	Cumulative inflammatory burden is associated with greater carotid plaque extent.	Direct PsA; longitudinal observational cohort [22].	Confounding and mediation are not excluded; vascular causality is unproven.
4–5	Subclinical atherosclerosis can be detected in PsA patients without overt cardiovascular disease.	Direct PsA imaging; observational imaging studies and meta-analysis [23,24,31].	Detection of existing disease burden; not evidence that universal screening improves outcomes.
5	Calculated event risk and measured plaque burden may be discordant.	Direct/mixed psoriatic-disease imaging and reclassification studies [25–28].	Discordance between constructs; not proof that a validated event-prediction equation has failed.
5	General-population risk equations have been externally evaluated in inflammatory disease, including PsA.	Direct PsA component within external validation study [29].	Model performance must be judged using validation metrics; no validated PsA-specific correction follows.
5	The historical EULAR 1.5 multiplication factor should not be automatically transferred to PsA.	Guideline-derived evidence [30].	The multiplier was principally developed for RA; no externally validated PsA-specific multiplier is established.
5	Different vascular territories provide non-identical information about subclinical atherosclerosis.	Direct PsA imaging; observational and cost-efficiency/multisite studies [32,33].	Detection/feasibility evidence; no incremental event-prediction or outcome benefit established.
5	Coronary atherosclerotic burden is detectable in mixed psoriasis/PsA cohorts and may carry prognostic information.	Mixed psoriatic-disease coronary imaging cohort [34].	Mixed population; does not establish a PsA-specific screening strategy.

Section	**Major claim retained in the review**	Evidence source/provenance and dominant design [ref.]	Limit of inference
6	NSAIDs and systemic glucocorticoids warrant minimization of unnecessary chronic exposure; methotrexate has generally reassuring observational cardiovascular signals.	General cardiovascular plus broader inflammatory-disease evidence; systematic review/meta-analysis and observational evidence [35,37].	No PsA-specific MACE estimate, causal cardioprotection, or comparative cardiovascular ranking can be inferred.
6	TNF-inhibitor exposure has been associated with lower event rates or more favorable subclinical atherosclerosis indices.	Mixed psoriatic-disease and direct PsA observational evidence [37–39].	No adequately powered PsA cardiovascular-endpoint trial; not evidence of causal cardioprotection or superiority.
6	Biologic/targeted therapy randomized-trial syntheses have not demonstrated a large short-term MACE signal.	Mixed psoriasis/PsA RCT meta-analyses [40,41].	Low event counts and short follow-up do not establish long-term equivalence or cardioprotection.
6	Some between-class differences in MACE hazards are reported in comparative observational PsA cohorts.	Direct PsA; weighted observational cohort [42].	Channeling bias and confounding by indication remain possible; no causal therapeutic hierarchy can be inferred.
6	JAK inhibitors require individualized cardiovascular and thromboembolic risk assessment.	Extrapolated RA safety RCT plus direct low-event PsA network meta-analysis [43,44].	RA effect sizes should not be directly transferred to PsA; no universal PsA class ranking is supported.
7	Conventional cardiovascular risk factors should be managed using validated regional risk assessment and established prevention guidance.	Guideline-derived/general cardiovascular evidence, with rheumatology comorbidity guidance [2,3,35,36,45].	Established prevention transferred to PsA care; not a PsA-specific score, multiplier, or treatment threshold.
7	Treat-to-target PsA management remains standard rheumatologic care, but cardiovascular-event prevention from treat-to-target has not been proven.	Guideline-derived plus direct PsA association data [2,3,17,22].	Association between disease activity and cardiovascular outcomes does not establish MACE reduction from a specific treat-to-target strategy.
Figure 1	The focused evidence map separates established prevention, direct PsA evidence, and unproven propositions.	Author interpretation based on the evidence categories above.	Conceptual organization only; not a guideline, prediction model, clinical pathway, or validated outcome-improving algorithm.

Abbreviations: CARMA, CARdiovascular in RheuMAtology; CV, cardiovascular; CVD, cardiovascular disease; EULAR, European Alliance of Associations for Rheumatology; ESC, European Society of Cardiology; JAK, Janus kinase; MACE, major adverse cardiovascular events; METS-IR, Metabolic Score for Insulin Resistance; NSAID, non-steroidal anti-inflammatory drug; PsA, psoriatic arthritis; RA, rheumatoid arthritis; RCT, randomized controlled trial; TNF, tumor necrosis factor.
